# Practical Notes and Formulæ

**Published:** 1884-09-20

**Authors:** 


### PRACTICAL NOTES AND FORMULÆ.

**Burn Ointment Unexcelled.**—

| | |
|---|---|
| Lead Plaster | 6 ounces |
| Spirits Turpentine and Linseed Oil of each | $1\frac{1}{4}$ ounces |
| Oil Origanum pure and Tinc. Opium of each | 6 dra'ms |
| Glycerine | 4 dra'ms |

Melt the lead plaster, then add the other ingredients, and stir till cool. For ulcerated condition of wounds, add to above amount one-half drachm acid carbolic. Apply to burns, scalds, etc., by spreading on cotton batting or cloth, to the thickness of one-eighth inch, renewing as often as ointment becomes dry; then scraping off only the upper layer and adding fresh ointment. No other treatment is needed until the wound is fully healed, after which no scars will be left, and immediate relief is given.—*Pharmacist and Chemist.* [N. B. If the denuded surface is extensive too much opium may be absorbed.—ED. RECORD.]

**Painless Escharotics.**—From the St. Louis Druggist we learn that Esmarch's painless caustic powder, for the removal of warts, tumors, etc., is composed of:

| | | |
|---|---|---|
| ℞ | Arsenious acid | part 1 |
| | Sulphate of morphia | part 1 |
| | Calomel | parts 8 |
| | Pulv. gum arabic | parts 48 |

This is to be sprikled on the cuticle daily. The surface should be denuded either with the knife or a blister. Canquoin's paste, for the same purpose, is made, according to M. Charles, by the following formula:

| | | |
|---|---|---|
| ℞ | Chloride of zinc, fused | parts 10 |
| | Alcohol, 60 deg. | parts 2 |
| | Wheat flour | parts 15 |

Rub the zinc chloride to a fine powder, add the alcohol, rub again and incorporate the flour, strongly pressing with the pestle. As soon as the paste is homogeneous, spread with a roller or bottle into sheets about one-eighth of an inch thick, and after a few hours put into a well-corked bottle.

Latour's nitro-chloride of zinc paste, a most excellent escharotic, is made by dissolving 50 parts of chloride of zinc and 100 parts of nitrate of zinc in 80 parts of water. The solution is made by the aid of heat. When it cools, add to each 100 parts of the fluid 75 parts of wheat flour, and incorporate as in Canquoin's paste.—*Med. and Surg. Reporter*, July 12, 1884.

**Ringworm.**—Two or three applications of the following, at intervals of eight or ten days, will frequently effect a cure:

| | | |
|---|---|---|
| R | Iodine | ʒ ij |
| | Kerosene oil | ℥ j |

M. To be applied with a firm brush. Dr. R. W. Taylor reports the best results from a paint composed of tincture of myrrh, with four grains of corrosive sublimate to the ounce.—*L. H. Washington, M. D., in St. Louis Medical Journal.*

**Hall's Solution of Strychnia.—**

| | | | |
|---|---|---|---|
| ℞ | Strychnia cryst | grs. | 6 |
| | Alcohol | fl.-℥ | 7½ |
| | Water | fl.-ʒ | 4 |
| | Acetic acid | fl.-ʒ | 4 |
| | Comp. tr. card | fl.-ʒ | 4 |

M. Ft. solut. Dose, 20 to 30 drops.—*Nat. Drug.*

**Fruit-Preserving Compound.**—A correspondent of the Druggist, of Chicago, gives the following recipe for preserving fruit without cooking:

| | | | |
|---|---|---|---|
| ℞ | Boracic acid | grs. | 192 |
| | Salicylic acid | " | 192 |
| | Carbonate soda | " | 192 |
| | White sugar | ℥ | 6 |
| | Water | ℥ | 12 |

Dissolve, and add sufficient water to make 8 pints.—*Nat. Drug.*

**Stye.**—If the stye should be very painful and inflamed, a small warm poultice of linseed meal, or bread and milk, should be laid over it, and renewed every five or six hours, and the bowels acted upon by purgative draughts. When the stye appears ripe it should be opened, and a little of the following ointment may be smeared over it twice a day:

| | | | |
|---|---|---|---|
| ℞ | Spermaceti | ʒ vj | |
| | White Wax | ʒ ij | |
| | Olive Oil | ℥ iij | M. |

Sometimes a stye may be cured by dipping a feather in the white of an egg and passing the feather lightly over the inflamed surface.

As a means of "backing" a stye, Dr. J. P. McGee used to advantage the following: ℞. Fluid Extract Belladonna, drops, iij; Pure Water, ℥, ij. M. Sig. A teaspoonful every hour.

Dr. L. Fitzpatrick writes to the Lancet that he has never seen a single instance in which the stye continued to develope after the following treatment had been resorted to: The lids should be held apart by the thumb and index finger, or a lid retractor, if such be at hand, while the tincture of iodine is painted over the inflamed papillæ with a fine camel's hair brush. The lids should not be allowed to come in contact until the part touched is dry. A few such applications in the twenty-four hours are sufficient.—*St. Louis Medical Journal.*

**Cholera Mixtures.**—The recent excitement about cholera has caused a demand at the drug stores for popular remedies. The following are the formulas for some of the principal ones:

SQUIBB'S CHOLERA MIXTURE.

℞ Chloroform .......... parts 3,<br>

Tr. opium .......... " 8,<br>

Spr. camphor .......... " 8,<br>

Tr. capsicum .......... " 8,<br>

Alcohol .......... " 13.<br>

Mix. Dose, 1 fluid-drachm.

ASIATIC TINCTURE FOR CHOLERA.

℞ Powd. opium .......... ℥ j,<br>

Camphor .......... ℥ j,<br>

Oil of cloves .......... fl. ℥ j,<br>

Powdered capsicum .......... ℥ j,<br>

Hoffman's anodyne .......... O j.<br>

Macerate two weeks and filter. Dose, 20 to 60 drops.

THIELEMAN'S CHOLERA DROPS.

℞ Oil of peppermint .......... fl ℥ j,<br>

Alcohol .......... fl ℥ viij,<br>

Tinc. opium and saffron .......... fl ℥ iij,<br>

" ipecac .......... fl ℥ viij,<br>

" valerian .......... fl ℥ 13½.<br>

Mix. Dose, 1 to 2 fluid-drachms.

LONDON BOARD OF HEALTH CHOLERA MIXTURE.

℞ Aromatic powder .......... ʒ iij,<br>

Aq. ammonia .......... fl ʒ iij,<br>

Tinct. catechu .......... fl ʒ x,<br>

" cardamom comp .......... fl ʒ vi,<br>

" opium .......... fl ℥ j,<br>

Chalk mixture, q. s. .......... fl ℥ x.<br>

Mix. Dose, 1 ounce.

SPARKMAN'S CHOLERA MIXTURE.

℞ Camphor .......... ʒ j,<br>

Kino .......... ℥ ij,<br>

Catechu .......... ℥ i-ij,<br>

Powdered cinnamon .......... ℥ ij,<br>

" cloves .......... ℥ j,<br>

" capsicum .......... ℥ ij,<br>

Brandy .......... q. s.

Moisten the powders with brandy, pack in a percolator, macerate forty-eight hours and percolate eighteen fluid-ounces. To this add:

Tinc. opium .......... fl ʒ xx,<br>

Chloroform .......... fl ℥ j.

Dose, 60 drops.—*National Druggist.*

**Phthisis.**—"In the treatment of high temperature of phthisis in Florida," says Dr. T. P. Miller, "the salicylate of sodium, in doses of grs. xvj to grs. xxiv, had been found to be the most serviceable antipyretic. When diarrhœa was present, from one-fourth to one-half a grain of morphia was added to each dose of the salicylate. The antipyretic should be given during the remission of the fever, and shortly before the exacerbation.

To relieve nausea and vomiting the following prescriptions are used according to circumstances:

℞ Acidi carbolici.................................ʒ j,

Tinct. iodini.................................ʒ ij.

M. Sig. Three drops in water, before food, three times a day; or

℞ Strychniæ.................................gr. j,
Acid. nitromuriat. dil.................................ʒ ss.
M. Sig. From four to eight drops, given as in the previous case.

**Treatment of Chronic Adenitis.**—An exchange publishes the following formulæ:

℞ Lard.........................30 grammes (say ℥ j)
Ammonium chloride............. 5 grammes (ʒ jss)
Camphor......................... 2 grammes (ʒ ss)
M. To be applied night and morning.

℞ Potassium iodide................. 1 gramme (gr. 15)

Distilled water ............... 5 grammes (ʒ jss)

Extract of hemlock .............. 2 grammes (ʒ ss)

Lard, or vaseline................30 grammes (℥ j)

M.

℞ Calomel, Acetate of lead, } aa............ 3 grammes (gr. 45)
Lard..............................20 grammes (ʒ v)
Camphor......................... 5 decigrammes (gr. 8)
M.—*Med. and Surgical Reporter.*

**For Catarrh.**—

℞ Carbolic acid.................................gtt. xx,
Iodine.................................gtt. xv,
Chloroform.................................gtt. xv.

A few drops of this mixture are to be heated over a spirit lamp in a test tube, the mouth of which is to be applied to the nostrils as soon as the liquid vaporizes: the operation is to be repeated after an interval of two minutes, when the patient will deliver a number of vigorous sneezes, and then his troublesome symptoms will quickly disappear.—*Ex.*

